# Sampling rate dependence of correlation at long time lags in BOLD fMRI measurements on humans and gel phantoms

**DOI:** 10.3389/fphys.2013.00106

**Published:** 2013-05-20

**Authors:** Kaare B. Mikkelsen, Torben E. Lund

**Affiliations:** ^1^Department of Physics and Astronomy, Science and Technology, Aarhus UniversityAarhus, Denmark; ^2^Center of Functionally Integrative Neuroscience, Aarhus University HospitalAarhus, Denmark

**Keywords:** fractional Gaussian noise, BOLD fMRI, Hurst exponent, edge effects, scanner artifacts

## Abstract

The aim of this study is to investigate the effects of sampling rate on Hurst exponents derived from Blood Oxygenation Level Dependent functional Magnetic Resonance Imaging (BOLD fMRI) resting state time series. fMRI measurements were performed on 2 human subjects and a selection of gel phantoms. From these, Hurst exponents were calculated. It was found that low sampling rates induced non-trivial exponents at sharp material transitions, and that Hurst exponents of human measurements had a strong TR-dependence. The findings are compared to theoretical considerations regarding the fractional Gaussian noise model and resampling, and it is found that the implications are problematic. This result should have a direct influence on the way future studies of low-frequency variation in BOLD fMRI data are conducted, especially if the fractional Gaussian noise model is considered. We recommend either using a different model (examples of such are referenced in the conclusion), or standardizing experimental procedures along an optimal sampling rate.

## 1. Introduction

For over a decade, neurovascular dynamics, represented by Blood Oxygenation Level Dependent functional Magnetic Resonance Imaging (BOLD fMRI) data, have been sought described using the language of fractals, or “1/*f*^β^ signals.” Examining the topics investigated, e.g., aging in Wink et al. (2006), Alzheimer's Disease in Maxim et al. (2005), or autism spectrum disorder, in Lai et al. (2010), the ideal of this approach is evident—to obtain microscopic information, or “hidden knowledge,” about the structure and health of a living and functioning brain, using only a minimally invasive method such as fMRI. Very recently, (Anderson et al., 2013; Baria et al., 2013) have shown a relationship between the power at low frequency and the degree of local connectivity, proving that this endeavour still has much to offer.

In the majority of the above referenced studies, the method applied to characterize the low-frequency behavior of the dynamics is the use of the Hurst exponent (*H*), usually building on an underlying assumption of the signal being fractional Gaussian noise (fGn) [see Equation (1) in the appendix, or Mandelbrot and Van Ness (1968)]. This is arguably the main method within this field of research, dating back as far as Fadili et al. (2001).

Briefly stated, *H* is a parameter between 0 and 1 describing the degree to which different points in the same time series are correlated, based on their separation in time. An *H*-value of 0 means a series of alternating low and high numbers (because immediate neighbors are highly anti-correlated), *H* = 0.5 is white noise and *H* = 1 is associated with an essentially constant time series. The case *H* > 0.5, in which measurements distant in time may be quite highly correlated, is often termed “long memory” in the literature, a convention which we have adopted in this paper.

“Long memory,” or power-law behavior of the power spectrum, is a useful concept, describing signals whose auto correlation structures have fat tails, implying that measurements distant in time will still be correlated. While this behavior appears to be ubiquitous throughout nature (Haslett and Raftery, 1989; Peng et al., 1995; Stephenson et al., 2000; He et al., 2010), science is still struggling with the task of succinctly explaining how it appears. In the case of physiological time series, including BOLD fMRI, an effect of the problem is a difficulty in determining the cause of an observed change in *H* (which may be both relevant or artefactual: (Eke et al., 2000; Kiviniemi et al., 2009).

For readers familiar with fGn, it needs to be noted that this paper distinguishes between fGn and true 1/*f*^β^-models. While these models are often lumped together, to say that fGn has a power spectrum on the form 1/*f*^β^ is merely an approximation, a fact which in the tests performed here turns out to be crucial. This point will be further treated in the discussion.

Related to the above-mentioned question of determining the origin of long memory, (Herman et al., 2011) studied the 1/*f*^β^-model on data from high-field fMRI in anaesthetized and post-mortem rat, finding that most, but not all, long memory disappeared after the sacrifice. This study complements their finding, by addressing *H*-based studies. Additionally, this investigation is closer to the premises of the previous human studies, in that it uses exclusively a standard 3 T human scanner, both on phantoms and human subjects. In agreement with Herman et al. (2011) we identify a minor, though persistent, non-cerebral component to the long memory in cortex. Finally, based on the findings presented in this article, we suggest means of improving the study of long time correlation in the brain, to both increase reproducibility and decrease artefacts.

## 2. Materials and methods

### 2.1. Data acquisition

All measurements were done on a Siemens Magnetom TrioTim 3T scanner, using a 32-channel head coil. The sequence was of gradient echo planar type, with echo time 30 ms, voxel dimensions 3 × 3 × 3 mm and a total Field of View at 192 × 192 mm for each slice.

As the very short repetition time (TR, the time between consecutive measurements) occasionally used forced us to only record one-slice volumes, only a single slice was ever used from each volume, regardless of the size of the volume. When the volume consisted of several slices, the middle slice was always used. The exception to this one-slice rule was in motion correction in SPM8, where all available data was employed.

The flip angles (FA) were chosen as the Ernst angles for T1 = 1100 ms, being the FA which maximizes the signal for a given TR (Ernst and Anderson, 1966). In this context, T1 is the time constant characterizing how the magnetization recovers along the magnetic field after having been excited by the initial RF pulse. For further details on MRI techniques, see (Haacke et al., 1999).

#### 2.1.1. Phantom measurements

The phantoms were of varying shapes, from cylinders to half-spheres. TR-values were either 60, 180, or 2000 ms, with FA varying from 10 to 90°. The effect of this variation is described below. To ensure accurate estimates of *H*, the lengths of the time series were either 4096 or 2000 volumes. The T1 values of the phantoms were around 1100 ms.

#### 2.1.2. Human measurements

For all human measurements, the scanner performed prospective motion correction (Thesen et al., 2000). The TR value was 80 ms, and FA 20°. Due to the short TR-value, only a single slice was recorded for each volume.

The two test subjects used in the study were the authors themselves. As such, both of them were male, in good health, with no known mental or physiological illnesses. The subjects were instructed to lie still and think of nothing in particular during the measurement. Given the identities of the research subjects, an ethical board was not involved.

For ideal testing of reproducibility, each subject was scanned twice, only allowing a short break in between for a walk around the scanner room, to reduce the risk of the subject falling asleep and ensuring that the two scans were conducted under conditions as identical as possible.

Each time series consisted of 4096 volumes.

### 2.2. Analysis

All measurements were analysed using SPM 8 (Wellcome Trust Centre for Neuroimaging, 2001), including standard motion correction (Ashburner, 2000) (with the exception of single-slice volumes, for which motion correction through SPM8 is not possible). In this regard, this study is in line with the studies referenced in the introduction. In select cases SPM 8 was used to make affine transformations (rotation + stretching + translation) between each volume in a time series, using the inbuilt “Normalize”-feature (Ashburner and Friston, 2005).

**Figure A1:**
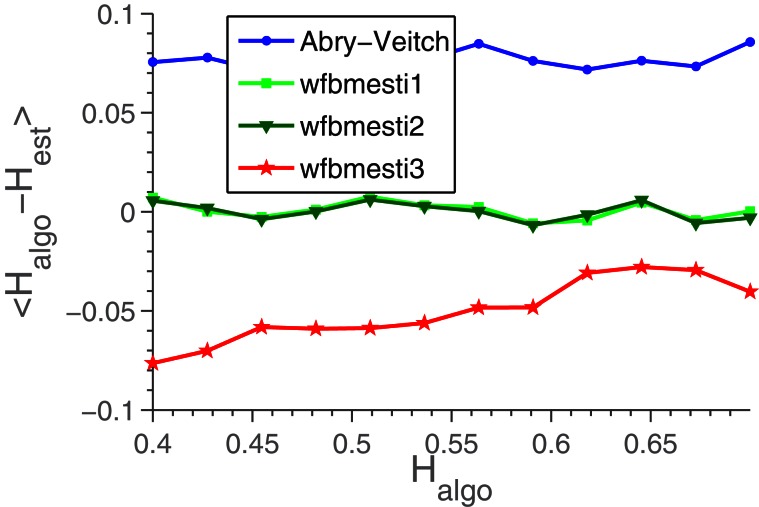
**The bias of four different estimators.** Each point is the average of *H*_est_ for 300 time series of length 1024, minus the *H*-value, *H*_algo_, used in generating the signals.

*H* was estimated using the first output of the MATLAB function wfbmesti, packaged with the Wavelet Toolbox. This estimator was also used by Kiviniemi et al. (2009), and was chosen based on a series of tests consisting of estimating *H* on artificially generated data, created using the algorithm: “circulant embedding of the covariance matrix,” described in Dietrich and Newsam (1997). Given that wfbmesti does not assume fGn statistics, but rather fractional Brownian motion [fBm, the integral of fGn, see Mandelbrot and Van Ness (1968)], the data was integrated (using the MATLAB function cumsum) before estimation. As can be seen from Figure A1 in the appendix, the chosen estimator achieved bias-free estimation. To underscore the fact that most of the *H*-values here are measured quantities, we will often be referring to *H*-estimates as *H*_est_.

**Figure 1:**
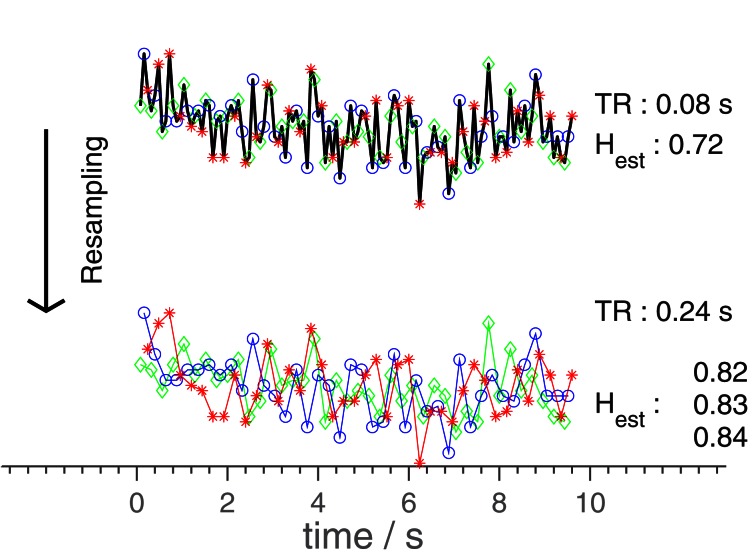
**Diagram illustrating the resampling-procedure.** The original time series is resampled, using only every third point, changing the effective TR from 0.08 to 0.24 s. The 3 *H*-values corresponds to the three different possible estimates. In general, an *n*-scaling of TR results in *n* different time series. As the original time series had a duration of 328 s, only a subset is shown here for clarity. The *H*-estimates were however calculated based on the entire time series (full duration), as was done in the analysis.

Besides through adjustments of the settings of the scanner, TR was changed artificially by removing volumes from the time series, to give as large a selection of TR-values as possible, while keeping the number of data points used in estimating *H* at at least 400. This practice is illustrated in Figure 1. As an *n*-order resampling can be done in *n* different ways, leading to *n* different *H*-estimates, the mean of these *n* estimates were used for each value of *n*TR. In the plots, data points originating from the same series of volumes (differing only by resampling) are connected by lines.

Except when otherwise stated, *H*_est_-values are averaged over the entire slice, including a thin layer of air around the object. This mask was preferred to one focusing on the edges of the object (1 voxel wide), as the scanned objects had different sizes requiring different masks, which could potentially introduce mask-dependent variation into the results.

## 3. Results

**Figure 2:**
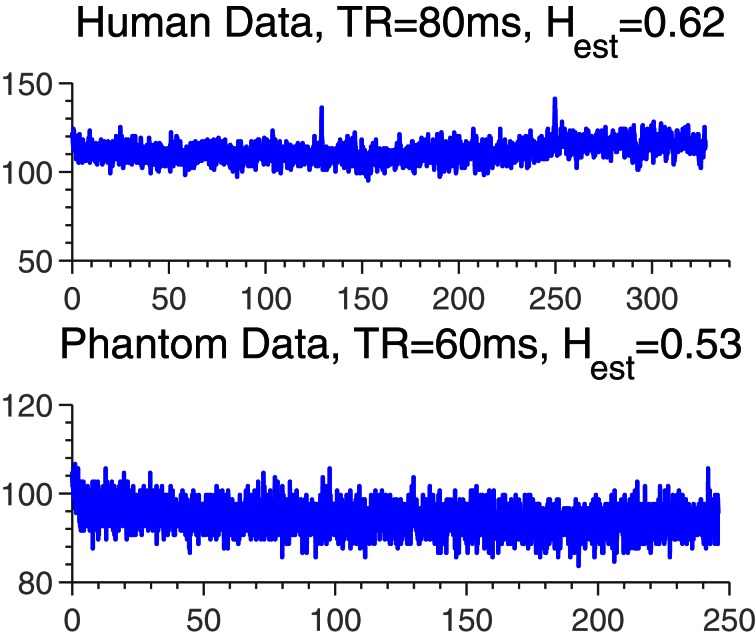
**Example time series from human and phantom data sets.** The *H*_est_-values are representative of the *H*_est_-distributions in either data set.

Time series were obtained as explained above. Example traces are plotted in Figure 2.

### 3.1. Phantoms

**Figure 3:**
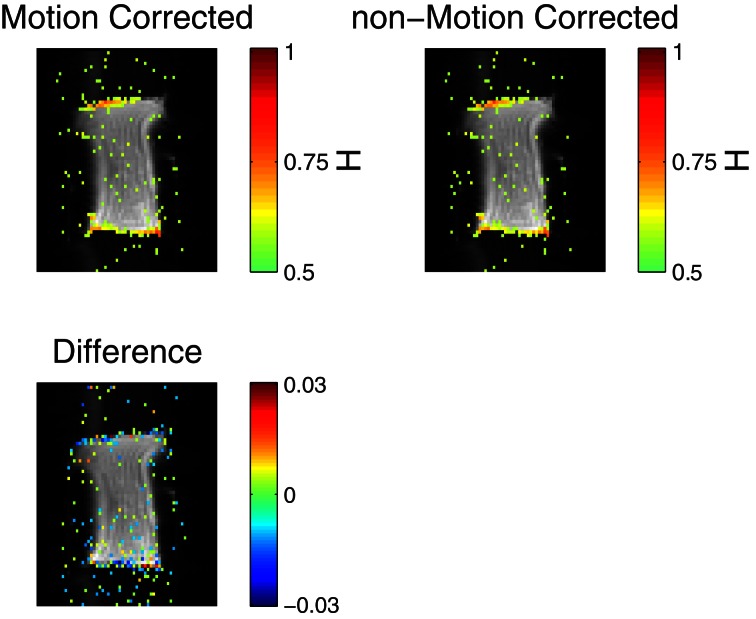
**(Top row) Voxels with *H*_est_ > 0.56 are superimposed on raw data showing the position of the gel phantom.** The color of a pixel represents the *H*_est_, as seen in the color bars. The top left image shows *H*_est_ for motion-corrected data, while the top right shows *H*_est_ for non-motion-corrected data. The bottom picture shows the difference between the two plots, for |*H*1 − *H*2| > 0.008. Note the small range on the colorbar. *TR* = 2000 ms, no artificial adjustment was made to TR. The time series consisted of 2000 volumes.

For all shapes and types of gel, it was found that for *TR* > 500 ms long memory was reliably detected on the edges of the phantom, when the FA was comparable to the Ernst angle. An example of this is seen in Figure 3, where voxels with *H*_est_ > 0.56 are colored in. It is clear that long memory is induced on the edges perpendicular to the phase encoding direction. In the figure is shown *H*_est_ with and without motion correction. It is apparent that the motion correction did not in any significant way change the amount of long memory.

**Figure 4:**
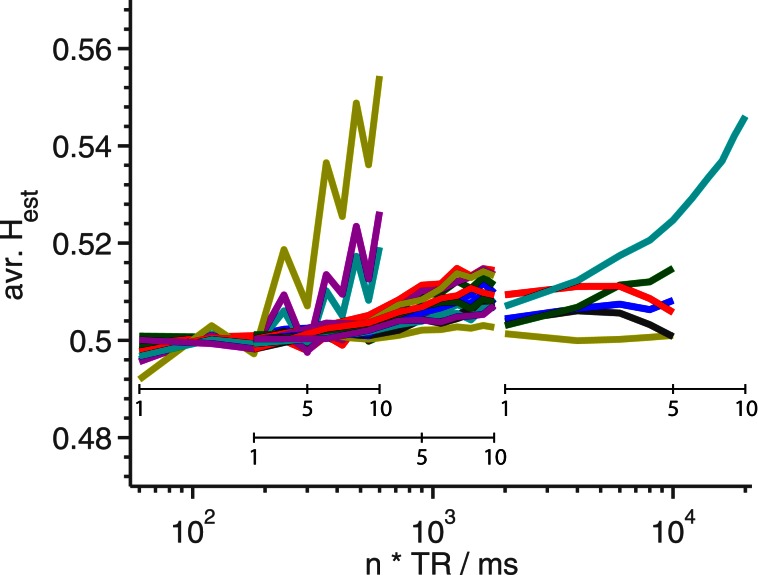
***H*_est_ averaged over the middle slice as a function of TR for phantom measurements.** Each line represents a time series which has its TR changed artificially by a factor of *n* as seen in Figure 1. For each line, the shortest TR corresponds to using all available data. We have found no obvious reason why a subset of the lines escape upwards around *TR* = 300 ms in such a marked fashion. The extra, smaller x-axes inside the plot show *n*-values for the three different TR-values represented in the plot.

In Figure 4 is shown *H*_est_ averaged over the entire slice, as a function of *n*TR, for all phantom data sets with FA close to the Ernst-angle.

We attempted to remove the artefactual long memory by applying an affine transformation between each volume in the time series, as an attempt at the most effective movement correction. However, this had no discernible effect.

**Figure 5:**
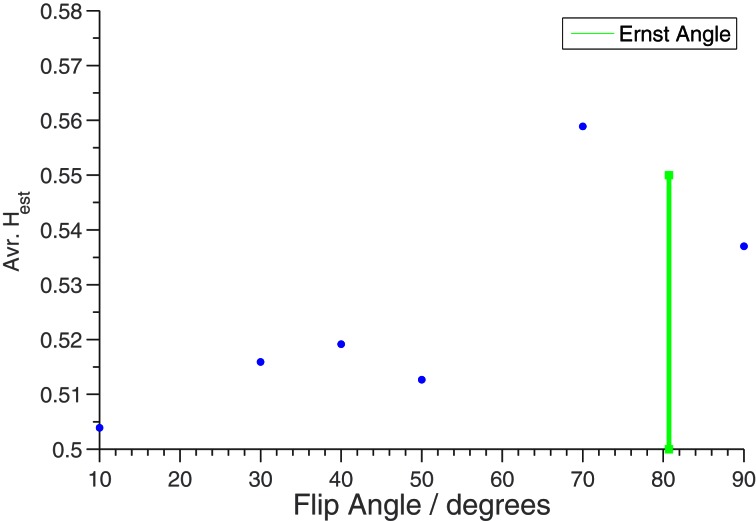
***H*_est_ averaged over middle slice as a function of flip angle for phantom measurements.** TR was 2 s. A mask was used to only include the edge of the phantom. The green bar shows the position of the Ernst Angle (80°) for T1 = 1100 ms.

In Figure 5 is shown the effect on the average *H*_est_ (restricted to the edge of the phantom) when the FA is varied. We see that the artefactual long memory largely disappears when the FA is changed from the Ernst angle. We interpret this effect to be caused by the rise in white noise level, indicating that the long memory effect is present in the measurements at the time of acquisition, and not induced in the subsequent post processing.

A series of almost identical phantom data sets were recorded, with increasing numbers of slices (but constant TR). From these, it was deduced that the artefactual long memory does not depend appreciably on scanner load (it was present in equal amounts for 1 slice, up to 35 slices).

Finally, we note that as the change in TR from 0.06 to 1 s is due to the resampling, and not in fact a change in measurement procedure, it seems unlikely that the decrease in long memory at low TR can be due to a rise in white noise level. The reason for this is that if at low TR the long memory was swamped by a too high white noise level, then that white noise would not disappear through a simple resampling, and thus equally rule out long memory at higher TR-values.

### 3.2. Human measurements

**Figure 6:**
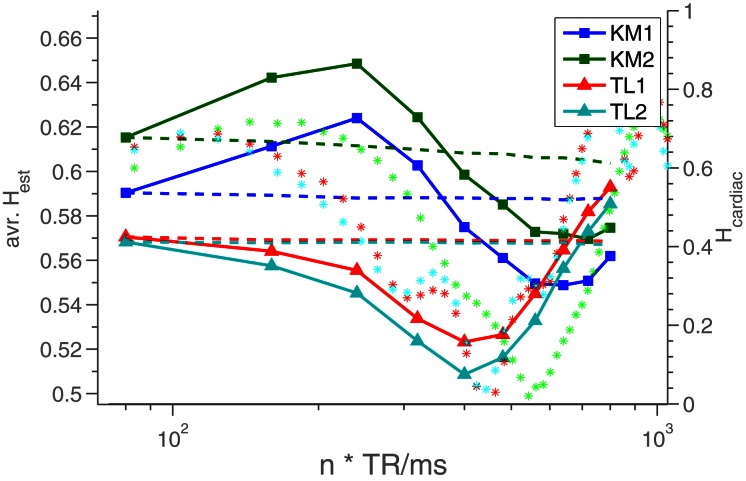
***H*_est_ averaged over the middle slice of the volume, for different TR values (solid lines).** KM and TL refer to the two different test subjects. The dashed lines are *H*-estimates from time series of equal length (relative to the resampled data), but constant TR, to demonstrate that the *H*_est_-fluctuations are not caused by the decreasing signal length. To do this, when TR was changed by a factor of *n*, the time series was cut into *n* pieces, which each yielded an *H*-estimate. The average of these *n* estimates was reported for the given TR. The stars (^*^) are *H*-estimates from resampled time series consisiting of mixtures of white noise and recorded cardiac data. These values can be read on the *right* y-axis.

In human measurements a somewhat different dependence on TR is seen, compared to the results from phantom measurements. In Figure 6 is seen the behavior of the average *H*_est_ as a function of *n*TR for two different human subjects each scanned twice. We see that while they each have relatively distinct *H*_est_(TR)-profiles, the answer to any question regarding “who has the most long memory” is dependent entirely upon the TR at which the measurement is done. For comparison, the plot also includes dashed lines showing *H*_est_ for time series of equal length, but constant TR. We see that the variation in *H*_est_ is not due to the decrease in time series length (caused by our artificial change of TR), but must be caused by the change in TR. This is further underscored by the fact that the two lines for A and B follow each other so closely - the intra-subject variability would clearly be much greater if the variation dictated by TR was due to decreasing signal length.

Part of the reason for this difference in behavior is likely that in humans, the scenario obviously is more complicated, given the existence of multiple additional low frequency noises sources. These include subject motion, pulse, respiration and variations in end tidal CO_2_ (Wise et al., 2004; Kiviniemi et al., 2009). To test this hypothesis, we created time series consisting of cardiac data, collected during the scans, and white noise, and tried resampling these. The *H*-estimates from these resampled datasets are also included in Figure 6, as ^*^'s. We see that they undergo oscillations similar to those of the human *H*_est_-values. The standard deviation of the white noise relative to that of the cardiac data was 1.2. This ratio was picked purely by trial and error, searching for behavior similar to what is seen for the real data.

## 4. Discussion

As shown in Figure 4, we find reason to recommend that long memory studies, especially those using the Hurst exponent, use as short a TR as possible to avoid artefactual long memory at transitions between materials. We think that the most likely explanation of the artefactual long memory is highly non-linear drifts in the scanner coordinate system. However, we have been unable to verify this theory, as neither the scanner load, nor the amount of motion correction, has had any impact on the presence of this artefact. As the origin of this phenomenon likely is tied to the equipment, we recommend that researchers intending to do studies of long memory in fMRI first look for similar artefacts using the intended equipment, and design their experiment accordingly.

We note that the human measurements were taken at very short TR, comparable to those in the artifact-free measurements, without a serious reduction in high-*H*_est_ voxels. In this light, we point out that while the study does not address the question of whether long memory in the BOLD fMRI measurements are related to neural dynamics, it does appear to rule out the possibility that it should be caused entirely by instabilities in the scanner. This is in line with the findings reported in Herman et al. (2011).

As shown in Figure 6, we find that *H*_est_ from human measurements fluctuates with TR, and that these fluctuations are different for different test subjects. We speculate that this variation, in part, is due to undersampling of physiological confounders. We have tested this hypothesis by creating time series as linear combinations of cardiac data and white noise, from which we can then obtain resampled *H*_est_-values, also included in the plot. We think that as a proof of concept, the comparison is quite good. It does not, however, tell us to which extent the long memory is due to the subject's pulse, and it is also clear that the chosen ratios of cardiac vs. white noise is somewhat arbitrary. In our experience, low-pass filtering an fGn-time series has very drastic effects on *H*_est_, meaning that any attempt to remove the cardiac contribution from the human data before estimation without influencing any non-artefactual fGn-component would be highly non-trivial and outside the scope of this article.

**Figure 7:**
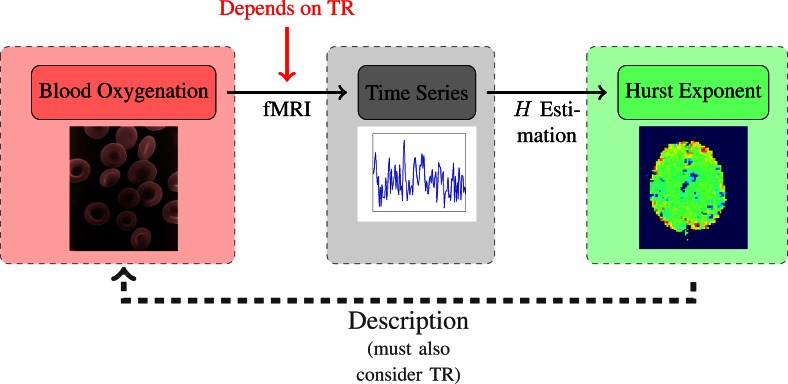
**Schematic of the relationship between TR and *H*_est_.** The crucial point is that prior to sampling, the continuous signal does not have a defined *H*-value.

Given the TR-dependence of the artefacts, and the TR-dependence of the human *H*-estimates, as seen in Figure 6, one might wonder what the optimal TR-value would be, and whether this could be identified by the degree to which it results in the “correct” *H*_est_ identical to the “true” *H*-value of the underlying process. However, as we show in the appendix, it is not possible to relate a specific *H*-value to a continuous process without first having properly defined how to discretize it. In itself, a continuous process, such as the BOLD response, does not have a well-defined *H*-value. Fractional Gaussian noise is not a continuous time process, and it can not describe continuous time dynamics. Instead, fGn can be used to describe a discretization of a continuous time process - in the case of this paper an fMRI time series (a set of discrete data points) representing the blood oxygenation (defined in continuous time). Which *H* is related to the continuous dynamics depends on how those dynamics are discretized, specifically what TR is used. This discordancy between the description and the phenomenon will persist for any choice of TR; while we are aware of the misconception that simply “sampling fast enough” will make an fGn time series continuous, this, regrettably, is not the case. As far as the definition of fGn is concerned, the data will always have time step “1”. We have tried to visualize the situation in Figure 7.

It is because of this discrepancy between continuous processes and their fGn-descriptions that we find it necessary to distinguish between the fGn-model and 1/*f*^β^-models. As shown in the appendix, *H*_est_ changes when an fGn-time series is resampled. In contrast to this, when dealing with a 1/*f*^β^-process, the absolute times of the measurements may go into the estimation of β, ideally reducing the effect of a resampling to a decrease of the Nyquist frequency, without changing the measured β. Given these very different behaviors, we hope that it is apparent to the reader why in this study a distinction is made, and why we recommend switching the fGn assumption to that of a continuous time model such as 1/*f*^β^.

We recommend solving this mismatch between model and phenomenon either through a change of model (away from fGn), or by fixing once and for all the time scale at which the blood oxygenation is studied (TR), the latter course of action having the effect of changing the studied phenomenon from a continuous time to a discrete time one. Of course, implicit in the latter course of action is the assumption that the interesting variation happens at the particular time scale chosen as TR.

Finally, it bears mentioning that as we find that the amount of long memory, as defined by Hurst exponents of fGn, in human measurements depends very much on TR (as shown in Figure 6), it seems unlikely that any new studies at very short TR would be comparable to older studies at long TR. Indeed, our results do question the validity of any hard-to-reproduce long TR results that might exist in the literature. This fact is related to the circumstance that the artifact identified in this paper appears at edges, which are a prominent feature of the cortex, which is the part of the brain where the long memory is generally detected (Maxim et al., 2005; Park et al., 2010). In relation to this, we point out that while it is true that Figure 4 is a comparison between two healthy individuals, and thereby does not directly predict the outcome of a comparison between healthy and ailing individuals, such as those studied in Maxim et al. (2005) and Lai et al. (2010), the difference between subjects KM and TL is, at its greatest, comparable to that seen between healthy and sick subjects in Maxim et al. (2005), while vanishing in other cases, making the results relevant.

It is interesting in this context to keep in mind that the literature does not demonstrate a consensus on what TR to use, but presents values varying by at least 80 % (e.g., Maxim et al., 2005 used both *TR* = 2000 and 1100 ms).

## 5. Conclusion

We find that in both humans and gel phantoms, *H*_est_ depends on TR. Based on the phantom measurements, we recommend using very short TR-values, but also that researchers test for edge-effects, similar to those documented in Figures 3, 4, before settling on a design for their experiment.

Based on the TR-dependence of *H*_est_ in human measurements, shown in Figure 6, we advice against comparing results from different studies using different TR-values.

We find, based on theoretical considerations described in detail in the appendix, that it is not possible to assign an *H*-value to the BOLD response without also settling on a sampling scheme. This is especially unfortunate, in that it seems a great hindrance in building any theoretical framework within which to attempt predicting *H*-values for different tissue-types or neurological diseases. It also means that it is not possible to use the TR-based variation in *H*_est_ to determine the appropriate TR.

Finally, we note that part of the issues here identified could possibly be avoided through a change of model, in favor of continuous time models such as 1/*f*^β^. Changing to a model compatible with continuous time dynamics would likely also be a great help for any future analytical work in bridging the gap between the microscopic model of blood oxygenation and macroscopic models, such as fGn. In this regard, we applaud the works presented in Park et al. (2010), Herman et al. (2011), Anderson et al. (2013), Baria et al. (2013), as being examples of studies using continuous time models. Especially the latter, for also exhibiting some of the theoretical work alluded to above.

### Conflict of interest statement

The authors declare that the research was conducted in the absence of any commercial or financial relationships that could be construed as a potential conflict of interest.

## Figures and Tables

**Figure A2:**
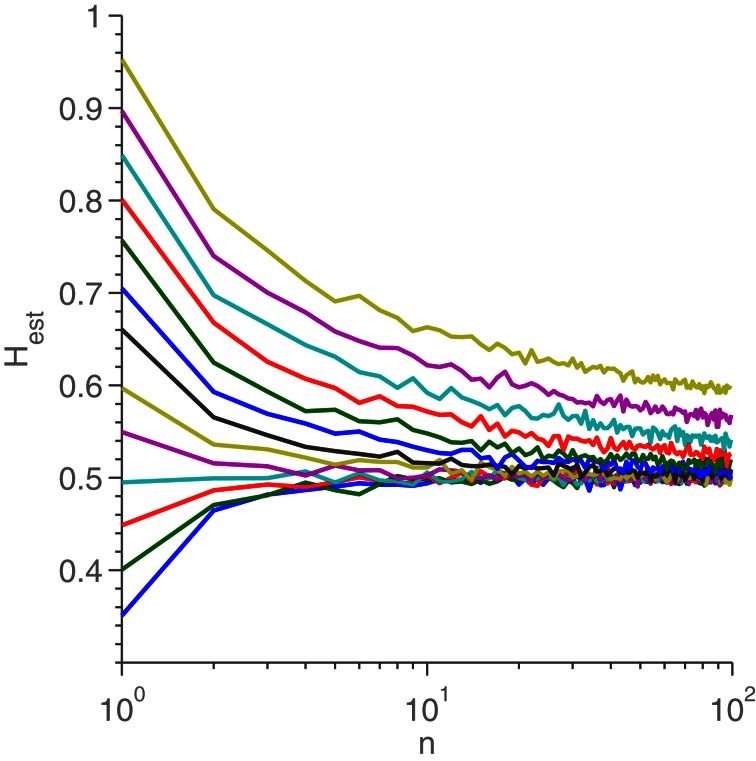
***H*_est_ as a function of *n* for different *H*_algo_.** For each line, *H*_algo_ can be read as the value for *n* = 1. Each point is the average of 100 *H*-estimations from different resampled fGn realizations. Each realization was only resampled once (instead of *n* times). After resampling, each time series was 1000 points long.

## Appendix

### A.1. Choice of estimator

In our test of estimators were used the three estimators bundled with the Wavelet Toolbox in MATLAB in the form of the function wfbmesti and our own implementation of the estimator described in Abry and Veitch (1998). In the case of wfbmesti what was passed to the estimator was in fact the cumulated sum of the signal, since that will transform fGn into fractional Brownian motion, which is what wfbmesti was designed to analyse. We were not able to obtain a MATLAB-compatible version of the estimator described in Maxim et al. (2005), which made it impractical to include it in the analysis. Based on the performance of the chosen estimator, we do not see this as a serious problem. In Figure A1 is seen the bias of each estimator, as a function of the *H*-value used in the signal-generating algorithm, *H*_algo_. The bias is defined as *H*_est_ − H_algo_ (*H*_est_ being the estimated value) averaged over 300 signals, each of length 1024. This signal length was chosen because it is short enough to be relevant experimentally, while long enough that the estimator-to-estimator variation was not dwarfed by the uncertainty caused by short time series. These considerations are backed up by the discussion in Park et al. (2010). Based on Figure A1, we decided to use wfbmesti-1.

### A.2. Effect of resampling on fGn

The auto-correlation structure, γ, of an fGn signal, *S*, is [Mandelbrot and Van Ness (1968)]:

(1) $$\begin{array}{l} \gamma (k)\text{​}=\text{​}\langle S(l+k)S(l)\rangle \text{​}=\text{​}\frac{\sigma }{2}\left({\left|k+1\right|}^{2H}+{\left|k-1\right|}^{2H}-2{\left|k\right|}^{2H}\right)\\ \;k:\text{lag},\;l:\text{index of measurement.} \end{array}$$

In the above, *σ* is the standard deviation of a particular measurement, or, in the case of *H* = 0.5, of the entire signal.

Changing TR means exchanging *S*(*i*) by some other set of numbers $\tilde{S}(j)$ which are different, but still exhibit the same general trends. As predicting the result of a measurement at a time *t*_*i*_ < *t* < *t*_*i*+1_, using measurements at times *t*_*i*_ and *t*_*i*+1_, is equivalent to a continuous extension of fGn, which is not possible, it follows that the assumption of fGn can not tell us what the likely outcome would be of performing the same experiment at a different TR-value. Except in one case, the one in which the new TR, *TR*_2_, is exactly an integer multiple of the original TR, *TR*_1_:

$${\text{TR}}_{2}=n{\text{TR}}_{1},n\text{ integer}$$

In this case, we retain every *n*'th data point, and throw out everything else. This means we can translate from one data set to another by scaling *i* by *n*:

(2) $$\begin{array}{l} \tilde{\gamma }(k)=\langle \tilde{S}(l+k)\tilde{S}(l)\rangle =\langle S(n\left\{l+k\right\})S(nl)\rangle \\ \;=\frac{\sigma }{2}\left({\left|nk+1\right|}^{2H}+{\left|nk-1\right|}^{2H}-2{\left|nk\right|}^{2H}\right) \end{array}$$

We see that it is impossible to bring (2) on the same form as (1), for *n* > 1. Therefore, the resampled time series is not, strictly speaking, fractional Gaussian noise. As such it is quite unlikely that, as *n* diverges from 1, the estimated *H*-value should stay the same. Simulations of the scenario, seen in Figure A2, confirms this prediction, yielding the quite reasonable result that *H*_*est*_ → 0.5 for *n* → ∞.

It should be mentioned that, while a great deal of effort has been expended in the attempt, we have been unable to come up with a way to define *S* “between the measurements” which did not result in wildly unpredictable *H*-estimates. Besides firmly denying any attempt at using non-integer *n*, it is also an excellent example of the difficulties in bridging the gap between a continuous phenomenon and a discrete description, as discussed in the conclusion.
